# Tumor-neutrophil crosstalk promotes *in vitro* and *in vivo* glioblastoma progression

**DOI:** 10.3389/fimmu.2023.1183465

**Published:** 2023-05-24

**Authors:** Dominique S. Rubenich, Priscila O. de Souza, Natalia Omizzollo, Mariana R. Aubin, Paulo J. Basso, Luisa M. Silva, Eloisa M. da Silva, Fernanda C. Teixeira, Gabriela F.S. Gentil, Jordana L. Domagalski, Maico T. Cunha, Kerolainy A. Gadelha, Leonardo F. Diel, Nicolly E. Gelsleichter, Aline S. Rubenich, Gabriela S. Lenz, Aline M. de Abreu, Giselle M. Kroeff, Ana H. Paz, Fernanda Visioli, Marcelo L. Lamers, Marcia R. Wink, Paulo V. Worm, Anelise B. Araújo, Jean Sévigny, Niels O. S. Câmara, Nils Ludwig, Elizandra Braganhol

**Affiliations:** ^1^ Programa de Pós-Graduação em Biociências, Universidade Federal de Ciências da Saúde de Porto Alegre (UFCSPA), Porto Alegre, RS, Brazil; ^2^ Laboratório de Células, Tecidos e Genes, Hospital de Clínicas de Porto Alegre (HCPA), Porto Alegre, RS, Brazil; ^3^ Departamento de Imunologia, Universidade de São Paulo (USP), São Paulo, Brazil; ^4^ Faculdade de Odontologia, Universidade Federal do Rio Grande do Sul (UFRGS), Porto Alegre, RS, Brazil; ^5^ Departamento de Ciências Morfológicas (ICBS), Universidade Federal do Rio Grande do Sul (UFRGS), Porto Alegre, RS, Brazil; ^6^ Serviço de Neurocirurgia, Hospital São José, Irmandade Santa Casa de Misericórdia de Porto Alegre (ISCMPA), Departamento de Cirurgia-Universidade Federal de Ciências da Saúde de Porto Alegre (UFCSPA), Porto Alegre, RS, Brazil; ^7^ Centre de Recherche du Centre Hospitalier Universitaire (CHU) de Québec, Université Laval, Québec City, QC, Canada; ^8^ Département de Microbiologie-Infectiologie et d’Immunologie, Faculté de Médecine, Université Laval, Québec City, QC, Canada; ^9^ Department of Oral and Maxillofacial Surgery, University Hospital Regensburg, Regensburg, Germany

**Keywords:** tumor associated neutrophils, glioblastoma, tumor microenvironment, cancer, neutrophil extracellular traps

## Abstract

**Introduction:**

The tumor microenvironment (TME) of glioblastoma (GB) is characterized by an increased infiltration of immunosuppressive cells that attenuate the antitumor immune response. The participation of neutrophils in tumor progression is still controversial and a dual role in the TME has been proposed. In this study, we show that neutrophils are reprogrammed by the tumor to ultimately promote GB progression.

**Methods:**

Using *in vitro* and *in vivo* assays, we demonstrate the existence of bidirectional GB and neutrophil communication, directly promoting an immunosuppressive TME.

**Results and discussion:**

Neutrophils have shown to play an important role in tumor malignancy especially in advanced 3D tumor model and Balb/c nude mice experiments, implying a time- and neutrophil concentration-dependent modulation. Studying the tumor energetic metabolism indicated a mitochondria mismatch shaping the TME secretome. The given data suggests a cytokine milieu in patients with GB that favors the recruitment of neutrophils, sustaining an anti-inflammatory profile which is associated with poor prognosis. Besides, glioma-neutrophil crosstalk has sustained a tumor prolonged activation via NETs formation, indicating the role of NFκB signaling in tumor progression. Moreover, clinical samples have indicated that neutrophil-lymphocyte ratio (NLR), IL-1β, and IL-10 are associated with poor outcomes in patients with GB.

**Conclusion:**

These results are relevant for understanding how tumor progression occurs and how immune cells can help in this process.

## Highlights

1) Glioma progression and malignancy is positively modulated by neutrophils.
2) Neutrophils induce glioma mitochondrial recovery.
3) Glioma-neutrophil crosstalk supports an immunosuppressive microenvironment.
4) Gliomas modulate neutrophil activation and increase lifespan.
5) Neutrophils affect cytokine profile production, correlating with poor prognosis.

## Introduction

Glioblastoma (GB) represents over 80% of the total cases of malignant gliomas and is confined to the central nervous system, rarely metastasizing to distant sites (1, 2). However, its histology shows that it is highly invasive and infiltrative with an acceleration of cellular and mitotic activity, angiogenesis, and necrosis (1). The tumor microenvironment (TME) of GB is characterized by an increased infiltration abundant in immunosuppressive cell populations attenuating the antitumor immune response (3). Interestingly, the infiltration rates of these immune cells are closely related to clinical outcomes (4). The permanence of immune cells, especially in the TME, supports an abnormal microenvironment of chronic inflammation – considered a hallmark of cancer (5, 6). In spite of leucocyte invasion, GB is considered a “cold tumor” due to its tall sums of regulatory B and T cells, as well as immunosuppressive myeloid cells (7).

Regarding the complexity of immune cell subpopulations, it was suggested in recent studies that neutrophils might play a significant role in stimulating disease progression in GB. Patients with increased levels of circulating neutrophils have increased neutrophils infiltration in the tumor tissue, which was linked to poor prognosis (8, 9). Neutrophils are myeloid components of the innate immune system, representing 50 to 70% of circulating leukocytes (10). They effectively participate in immune responses, using various mechanisms, such as phagocytosis, degranulation, and/or formation of neutrophil extracellular trap (NET) (11). Overall, neutrophils can be characterized by their phenotypic and functional plasticity as pro-tumor or anti-tumor cells (12). The controversy of the contribution of neutrophils to tumor progression has been used to explain their dual role in the TME (13).

The contribution of nonmalignant cells to tumor development and progression is now indisputable (14). The diffuse nature of the tumor, which infiltrates adjacent tissues and varies in character and location within the brain, result in the inability to resect the tumor completely. Despite the many studies, it is difficult to establish efficient immunotherapies against GB due to tumor evasion mechanisms (15). Therefore, effective therapies have yet to be discovered, which makes this tumor an important target for studies with different approaches. Neutrophils might be a potential target for better patient outcome (16). Thus, this study proposes that neutrophils promote GB progression after being reprogrammed by the tumor. We report the existence of GB-neutrophil bidirectional communication with direct impact on promoting an immunosuppressive TME.

## Materials and methods

### Material

The chemicals employed in this study are listed in **Supplementary Table 1**. All other chemicals and solvents were obtained from standard commercial suppliers with analytical grade standard and were used as received.

### Human cell culture

Human U87MG glioma cell line obtained from ATCC (American Type Cell Collection; Rockville, Maryland, USA) was grown in Dulbecco’s modified Eagle’s medium (DMEM – Sigma-Aldrich, USA) prepared with 8.4 mM HEPES, 23.8 mM sodium bicarbonate (NaHCO_3_), 0.1% fungizone, 100 U/L penicillin/streptomycin 0.5 U/L and 10% of heat-inactivated Fetal Bovine Serum (FBS – Gibco, USA), at final pH of 7.4. Cells were incubated at 37°C with 95% minimum relative humidity and 5% CO_2_.

### Primary neutrophil cultures

Peripheral blood was collected from healthy volunteers into EDTA blood collection tubes. Within less than 15 min after the donation, the blood was processed following the described protocol elsewhere (17). Briefly, the blood was entirely diluted with an equal volume of 2% Dextran (Sigma-Aldrich, USA) in saline solution and incubated for 20 min at room temperature (RT). Afterwards, leucocytes were collected and gently loaded on top of Histopaque-1077^®^ (Sigma-Aldrich, USA) with 1:2 volume proportion. Continuous centrifugation, at 428 *×* g, for 30 min, at RT, was used for separation of low-density leucocytes from high-density ones. The high-density fraction with polymorphonuclear cells (PMN) was further subjected to red blood cell lysis with Milli-Q water for 25 seconds and neutralization with HBSS 10x concentration. To remove the lysis product, the sample was centrifuged for 10 min, at 400 *×* g, and at RT. Isolated neutrophils (Nθ) were cultured in DMEM supplemented with 10% FBS. The institutional ethics review board of Hospital de Clínicas de Porto Alegre (n° 2.969.418) approved this study.

### 2D Glioblastoma-neutrophil co-culture

U87MG cells were seeded in 48-well plates (15 × 10^3^ cells/well) and cultured for 1.5 h before neutrophils were added in the ratios 1:30, or 1:60, (GB to Nθ, respectively). Co-cultures were incubated for 24, 72, and 120 h. Monocultures of GB and Nθ cells were applied as controls. The following assays were performed:

#### MTT assay

Soluble MTT [3-(4,5-dimethylthiazol-2-yl)-2,5-diphenyltetrazolium bromide] reduction assay to formazan crystals was performed as previously described (18). The absorbance was determined at 560 and 630 nm on a microplate reader (SpectraMax^®^ M3-Molecular Devices). Data were expressed as absorbance and estimated by the difference between 560 and 630 nm, and the MTT absorbance of U87MG cells were considered as the control (CRTL) group by 100%.

#### SRB assay

The sulforhodamine B (SRB) assay was used for cell density determination and it is based on the measurement of cellular protein content. U87MG monolayers were fixed with 10% trichloroacetic acid for 1 h, stained with SRB solution for 15 min, and the excess dye was completely removed by washing repeatedly with 1% acetic acid. The protein-bound dye was dissolved in 10 mM Tris base solution for optical density (OD) determination at 515 nm using a microplate reader (19). Data were expressed as percentage of CTRL.

#### Trypan blue assay

Viable and dead neutrophils were counted in a Neubauer chamber after trypan-blue staining (2%) as previously described (20).

#### Flow cytometry

To test cell viability, cells were stained with Live/Dead fluorescent dye – AmCyam 1:400 (Thermo Fisher, EUA). Cells were clustered by FSC x SSC, followed by doublets removal and selection of live cells only (**Supplementary Figure 1**). For mitochondria assay, cells were washed in PBS and stained with 200 nM MitoTracker Green (Invitrogen, Carlsbad, CA), 50 nM MitoTracker Deep Red (Invitrogen), and MitoSOX (5 μM). For glucose-uptake assay, after washing with PBS, cells were resuspended in 100 µL of RPMI1640 without bicarbonate, containing 5 mM glucose and 5 mM glucose fluorescent analogue, 2-(N-(7-Nitrobenz-2-oxa-1,3-diazol-4- yl)amino)-2-Deoxyglucose (2-NBDG), for 20 min at 37°C. After the incubation period, cells were washed and resuspended in PBS containing 2% FBS and immediately acquired in a flow cytometer. Samples were analyzed using a FACSCanto flow cytometer driven by BD FACS Diva software (BD Biosciences, San Diego, USA) and were processed using Flow-Jo software (FlowJo LLC, Ashland OR).

#### Glucose and lactate determination

Quantification of glucose and lactate in supernatants was performed using enzymatic systems (ref n° 133 and ref n° 138, Labtest), according to manufacturer’s recommendations. Absorbance was determined at 505 nm (glucose) and 550 nm (lactate) wavelengths in a spectrophotometer (Synergy™ Mx Microplate Reader; BioTek) coupled to Gen5™ software (BioTek). Results were expressed as mg/dL of culture supernatant normalized per mg of protein.

#### Cytokine cytometric bead array

The Human-Inflammatory Cytokine Based Assay (CBA) kit for IL-8, IL-1β, IL-6, IL-10, TNFα, and IL-12p70 (BD Biosciences, San Diego, CA, USA) was performed according to manufacturer’s recommendations.

### GB-neutrophil 3D co-culture

Three-dimensional spheroids were generated using the hanging drop technique (21). After the enzymatic dissociation of the U87MG culture, the cell suspension was diluted to a final concentration of 25×10^3^ cells per 30 µL. Each drop containing 30 µL was placed inside the cap of a 48-well plate previously treated with agarose 1.5% and filled with 300 µL of PBS 1× in order to prevent dryness of the drop (22). Rolling and falling off from drops were considered as failed procedure. Plates were kept immobile in incubators at 37°C and 5% CO_2_, during 4 days for the establishment of cell aggregates. After that, each well was filled to their maximum volume with DMEM supplemented with 10% FBS for the purpose of causing the sphere to fall gently into the well. Only one spheroid per well was cultured for more than 2 days. The co-culture procedure started with 7.5 × 10^3^/500 µL neutrophils for each spheroid to mimic initial tumor communication and the 3D media was totally removed and replaced with the one containing recently isolated PMNs. GB and Nθ cultured alone were considered the control of experiment. The following assays were performed:

#### Advanced tumor model

The neutrophil infiltrate assay aimed to mimic neutrophil communication with advanced tumors. During the assay, a “pool” of neutrophils was applied to the environment of an U87MG sphere. Thus, after removing the cell aggregate from the culture medium, 7.5 × 10^5^/500 µL of neutrophils were added to each tumor spheroid. The spheroids were incubated for 24 h and 72 h, at 37°C, and at 5% CO_2_.

#### Histopathologic evaluation of 3D cultures

The spheroids were fixed with 4% paraformaldehyde for 18 h and left immersed in PBS until histological processing. The protocol adapted from the agarose block was used as a 2% agarose template containing microwells for each bead (23). The mold was inserted into the histological cassette for processing in the OMA pv.85 histotechnical equipment (Instrumental OMA Ltda, São Paulo, Brazil). The material was dehydrated with a series of alcoholic solutions with increasing concentrations (50%, 70%, 90% methanol bath and four baths in 100% methanol, 1 h each), followed by the clarification process of 3 baths of xylene (1 h each) and impregnation with liquid paraffin (2 baths of 2 h each). Sections of 4 μm slides were performed on a Leica RM2255 microtome (Leica Microsystems, Inc., Wetzlar, Germany). The material obtained was collected with positively charged microscopy glass slides.

For hematoxylin and eosin (HE), staining slides were dewaxed with 3 baths of xylene (5 min each) and rehydrated in descending scale ethanol baths (1 min each). Staining was performed with hematoxylin monohydrate (Merck KGaA, Darmstadt, Germany) and yellowish eosin (Synth, Diadema, Brazil).

For immunohistochemistry, slides were submitted to the antigen recovery process, using a solution composed of 10 mM sodium citrate (pH 6.0) for Ki67. The samples were incubated with the respective recovery solutions at 98°C, for 30 min. The blockage of endogenous peroxidase activity was performed with 5% hydrogen peroxide solution (Merck KGaA), and nonspecific binding was blocked with 1% BSA (Sigma-Aldrich, St. Louis, Missouri, USA). Anti-Ki67 (1:100, Dako, Santa Clara, USA) antibody was incubated for 12 h, at 4°C. The detection kit (Dako, Santa Clara, USA) was used according to the manufacturer’s recommendations. The percentage of positively labeled cells for each sample was counted from the average of the total positives and negatives of five images per sample using ImageJ Software. The slides were analyzed in a blinded manner by a pathologist.

#### Time-lapse microscopy

The spheroids were submerged into an environment containing 10^6^ neutrophil, and time-lapse images were captured for a period of 20 h, at 10-min intervals, with camera (Axiocam mrn, Zeiss, Göttingen, Germany) attached to an inverted microscope (Axio Observer Z1, Zeiss, Göttingen, Germany). The sphere area from each time point was plotted and analyzed using ImageJ software. The growth of spheres was determined by comparing the initial and the final size of each spheroid and expressed as area (pixel^2^).

#### Annexin V/propidium iodide assay

Apoptosis/necrosis was evaluated using annexin V/propidium iodide (PI) staining followed by flow cytometry. Glioma spheroids produced and treated with neutrophils for 72 h and 120 h were collected from the four groups and dissociated. Cells were centrifuged at 300 × *g* for 5 min and washed twice with cold PBS. Afterwards, the cells were resuspended in binding buffer and incubated with PI and Annexin V-FITC (BD Biosciences Pharmingen, San Diego, CA) for 15 min, at RT. A total of 10,000 events were collected and analyzed by flow cytometry (FACSCanto, BD). As tumor and Nθ cells exhibit highly distinct size and complexity patterns, the neutrophil and tumor cell population gate were based on scatter plot. Initial apoptotic cells were taken as Annexin V-positive/PI-negative, while late apoptotic cells were taken as Annexin V-positive/PI-positive and necrotic cells were taken as Annexin V-negative/PI-positive cells. Live cells were taken as Annexin V-negative/PI-negative.

### *In vivo* tumorigenicity experiment

The animal ethics committee of Hospital de Clínicas de Porto Alegre approved our studies with animals (n° 2020-0708). Male and female BALB/c-nude mice (n=40; specific-pathogen free; 8-12 weeks old; 22-26 g) were housed at 20-24°C with a relative humidity of 40-60%, consuming an average of 3-5 g of food and 5-10 mL of water per day, with a light/dark cycle of 12 h/12 h. The mice were divided into four groups with ten nude mice in each group (5 male, 5 female). The tumorigenicity experiments were performed within a laminar flow cabinet. The following analysis consisted of a subcutaneous heterotopic model with the implantation of 10^6^ U87MG with increasing concentrations of neutrophils (3%, 10%, and 20%) injected into the right flank of mice. Cell suspension (100 μL) and Matrigel (Sigma-Aldrich, E1270) (100 μL; 5-6 mg/mL) were applied to the animals subcutaneously (final volume applied 200 μL). The control group received only tumor cells. The two distances (diameters) at right angles were measured every two days, i.e., the width (w) – which is the greatest transverse diameter, and the length (l) – the greatest longitudinal diameter) until tumors reached the endpoint of 2000 mm^3^. At the end of the experiments, tumors were fixed with 4% paraformaldehyde and processed for HE histological staining and immunohistochemical for Ki67 as described above. The slices were analyzed by a pathologist in a blinded manner.


$$Volume\;\left(mm^{3}\right)=\left(\frac{w^{2}*l}{2}\right)\times 1000$$

### *In vivo* neutrophil maturation status

Neutrophils extracted from the bone marrow of C57/BL6 mice according to the protocol described by Liu and colleagues (24). Therefore, murine Nθ were challenged with GL261 cells (mouse glioma cell line obtained from ATCC) following the same conditions as U87MG + Nθ described earlier for 24h. CD11b^+^ cells (1:400) were further divided into the following groups: mature: Ly6G^high^ (1:400), CXCR4^-^ (1:100), CXCR2^+^ (1:100); immature: Ly6G^low/mid^, CXCR4^+^, CXCR2^low^; aged: Ly6G^high^, CXCR4^+^, CXCR2^-^ (25). Animal studies were approved by the ethics committee of UFCSPA and USP (n° 278/20 and n° 6769171122, respectively).

### Sytox green: NETs assay

Aliquots of 10^6^ neutrophils/mL in HBSS/Ca^+2^ (1 mL) was prepared in microtubes following the planned groups. The sytox green reagent (Invitrogen) was added to each tube at a final concentration of 0.2 µM. Cells exposed to HBSS 1× or Triton 100× were applied as negative and positive controls, respectively. U87MG conditioned media was produced according to the following parameters: after reaching 85% confluency in T75-flask, 10 mL of DMEM with 0.5% or 10% FBS were kept for 72 h, at 37°C, 5% CO_2_. PMA (100 nM) and LPS (1 μg/mL) groups were performed as control. PMNs were incubated for 3 h and 200 µL of each condition was transferred to a separate well of a 96-well black plate. Fluorescence was quantified at excitation/emission wavelengths of 488/520 nm with a microplate reader (26). The results were expressed as relative fold change by dividing the average of the experimental condition by the average of the untreated condition (negative control).

### *In silico* TCGA analysis

Data from GBM dataset (n = 172) was downloaded from the Cancer Genome Atlas (TCGA; https://gdc.nci.nih.gov). Gene expression profiles were analyzed for glioma poor prognosis markers (IDH and MGMT), cell signaling pathways (TGFβ1 and NFκB1), epithelial-mesenchymal transition (CDH1 and CDH2), mitochondria activation (FOXO3 and ATF5) and neutrophil markers (MPO and ELANE) using the XENA browser (UC Santa Cruz) (27). Pearson test correlation matrix was performed to analyze the strength of association. Correlation was indicated as follow: small is 0.1-0.3 (positive) and −0.1 to −0.3 (negative); medium is 0.3 to 0.5 (positive) and −0.3 to −0.5 (negative); and large is 0.5 to 1.0 (positive) and −0.5 to −1.0 (negative) for the same gene set.

### Glioma patients

Medical records of 43 patients who underwent surgery for glioma resection at Irmandade Santa Casa de Misericórdia de Porto Alegre (ISCMPA) from 2019 to 2022 were prospectively reviewed. Blood was collected at time of surgery. Patients were divided into two groups based on histopathological classification (glioblastoma vs. non-glioblastoma). The following variables for each patient were studied: sex, age, and the number of leucocytes, neutrophils, lymphocytes, and monocytes in the circulation. The neutrophil to lymphocyte ratio (NLR) was estimated. The institutional ethics review board of ISCMPA (n° 3.204.937) approved this study. Voluntary donors signed the informed consent form.

### Statistical analysis

Data are expressed as mean and standard deviation (SD) or as standard error of the mean (SEM) when appropriate of at least three independent experiments and were subjected to two-way analysis of variance (ANOVA) followed by Tukey–Kramer *post-hoc* test (for multiple comparisons) or One-way ANOVA, Bonferroni’s test or Student’s *t-*test, when appropriate. Differences between mean values were considered significant when *P* < 0.05.

## Results

### Glioma-neutrophil crosstalk induces tumor cell proliferation

**Figure 1:**
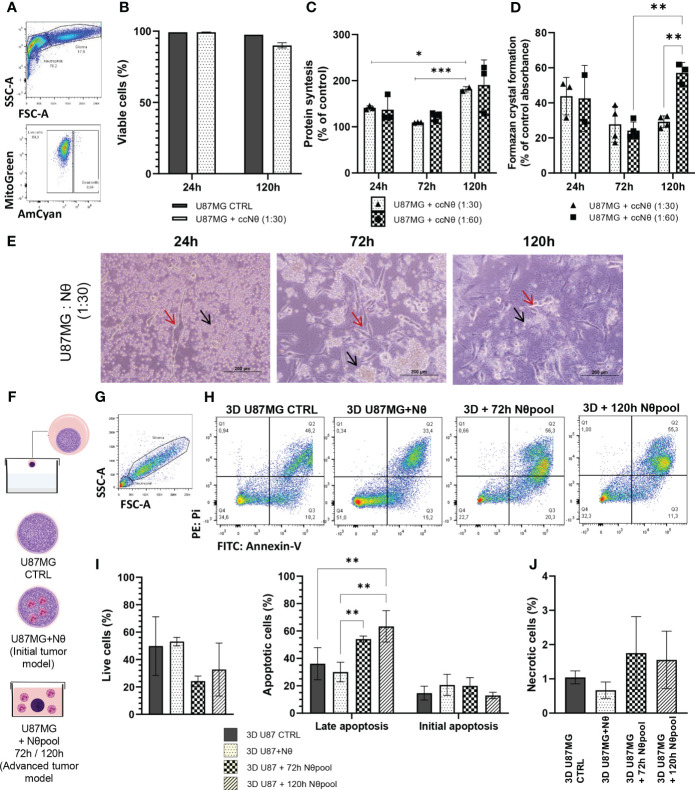
**(A)** Gating strategy to select living glioma cells (U87MG), by FSC x SSC, followed by selection of live cells only. **(B)** Quantification of U87MG live cells percentage. **(C)** SRB assay performed on a monolayer of U87MG with or without neutrophil coculture (ccNq) after 24 h, 72 h, and 120 h. The data is expressed by cell growth considering U87MG control result as 100% in each time. **(D)** MTT assay performed on a monolayer of U87MG with or without neutrophil co-culture after 24 h, 72 h, and 120 h. The data is expressed by MTT percentage considering U87MG control result as 100% in each time. **(E)** Representative images of glioma and neutrophil culture. Red arrow glioma and black arrow neutrophil. **(F)** Hang drop technique protocol. **(G)** Gating strategy to select glioma cells (U87MG) from 3D culture. **(H)** Gating strategy for initial apoptotic cells were taken as Annexin Vpositive/PI-negative, while late apoptotic cells were taken as Annexin V-positive/PI-positive and necrotic cells were taken as Annexin V-negative/PIpositive cells. Live cells were taken as Annexin V-negative/PI-negative. Apoptosis measurement using Annexin V/PI double staining of 3D U87MG culture. Bar graphs representing the percentage of live cells and apoptotic cells **(I)** and necrotic **(J)** with or without neutrophil. Two-way ANOVA, multiple comparisons, Tukey test, in which a = 0.05 and *P < 0.05, **P < 0.005, ***P < 0.001. Error bars are mean ± SD.

To assess the bidirectional communication between cultured glioma cells (U87MG) and human neutrophils, we performed co-culture studies. Firstly, the viability of U87MG cultures was measured by flow cytometry in the presence and absence of neutrophils (**Figure 1**). U87MG viability was sustained regardless of neutrophil interaction (**Figures 1A, B**). However, U87MG protein synthesis (**Figure 1C**) and MTT absorbance (**Figure 1D**) suggests that, in the first 24 h and 72 h of culture, neutrophils negatively affect the viability of U87MG cells. After 120 h of neutrophil-glioma co-culture, tumor growth seemed to extrapolate the previous pattern. Moreover, protein synthesis was proportional to MTT absorbance, indicating a metabolic recovery. We also observed the cellular interactions of U87MG (red arrow) and neutrophils (black arrow) by light microscopy (**Figure 1E**), and neutrophil distribution changed over time, clustering around tumor cells. Furthermore, 3D culture was used to test neutrophil infiltration capability and tumor proliferation. **Figure 1F** shows the experimental groups of the 3D culture experiment, with U87MG spheres as CTRL, U87MG with neutrophils sphere formation representing initial tumor, and U87MG spheres immersed on neutrophil pool representing advanced tumor. Therefore, to investigate the effect of neutrophils on apoptosis of U87MG 3D culture, the cells were double-stained with Annexin V and PI, prior to detection using flow cytometry (**Figures 1G, H**). Quantitative analysis demonstrated a slight reduction in cell viability, followed by an increase in U87MG cells undergoing late apoptosis in the presence of neutrophils in advanced tumor 3D model (**Figure 1I**), in addition to augmentation of necrotic death (**Figure 1J**). Given data suggests interaction with neutrophils stimulates glioma proliferation over time in the 2D culture model, while late apoptosis was detected in the advanced tumor spheroids.

### 3D culture model indicates that neutrophils alter glioma cell organization

**Figure 2:**
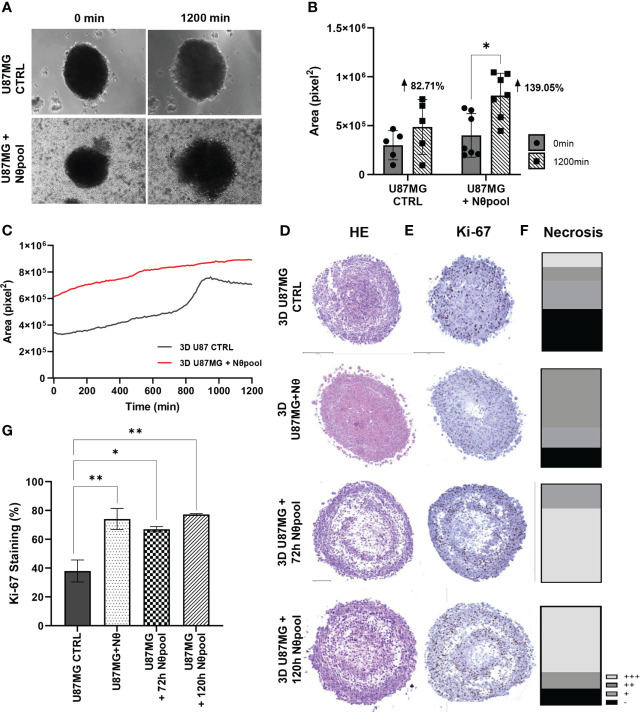
**(A)** Neutrophil interaction with 3D U87MG promotes sphere growth. 3D U87MG culture were placed with 10^6^ neutrophils and performed time-lapse analysis during 20 h. **(B)** Initial (0 h) and final (20 h) areas were compared between groups **(C)**, and the mean tumor growth pattern was also observed. **(D)** Representative image from HE, **(E)** Ki-67 staining and **(F)** necrosis extent analysis. **(G)** Ki-67 quantification representing the percentage of positive cells. Two-way ANOVA, multiple comparisons. Tukey test, in which a = 0.05, *P < 0.05 and **P < 0.005. Error bars are mean ± SD.

Given the results regarding tumor cell proliferation, we hypothesized that neutrophils have further tumor-promoting effects. Therefore, we evaluated the growth of 3D spheres for 20 h using time-lapse technology. Glioma spheres with neutrophil pool expanded significantly faster (*P* < 0.05; **Figures 2A, B**) and showed a distinct growth pattern over time (**Figure 2C**). While glioma spheres seemed to grow linearly in the presence of neutrophils, the U87MG control showed a peak between 800 and 1000 min and stabilizes itself. Thus, we conducted a histopathological analysis, including HE staining of spheres (**Figure 2D**) and Ki67 staining to assess cell proliferation (**Figure 2E**). These experiments revealed a shift of glioma cell morphology. While CTRL cells were characterized by a fusiform cell shape, groups with neutrophils appeared as small and homogeneous cells (**Supplementary Table 2**). The quantification of Ki67 staining revealed a significant increase of cell proliferation in neutrophil-treated groups (*P* < 0.01; **Figure 2G**), especially in the border zones of the spheres (**Figure 2E**), along with slightly extension of the necrotic area (**Figure 2F**). The neutrophil infiltration in the spheroids was accessed by CD45 immunostaining and a small percentage of positive cells was detected (**Figure S1**). Taken together, these data are in line with the results obtained using 2D culture model and reinforce the role of neutrophils as promoters of GB cell proliferation.

### Glioma-neutrophil crosstalk attenuates initial tumor growth, but stimulates growth pattern in advanced tumors

**Figure 3:**
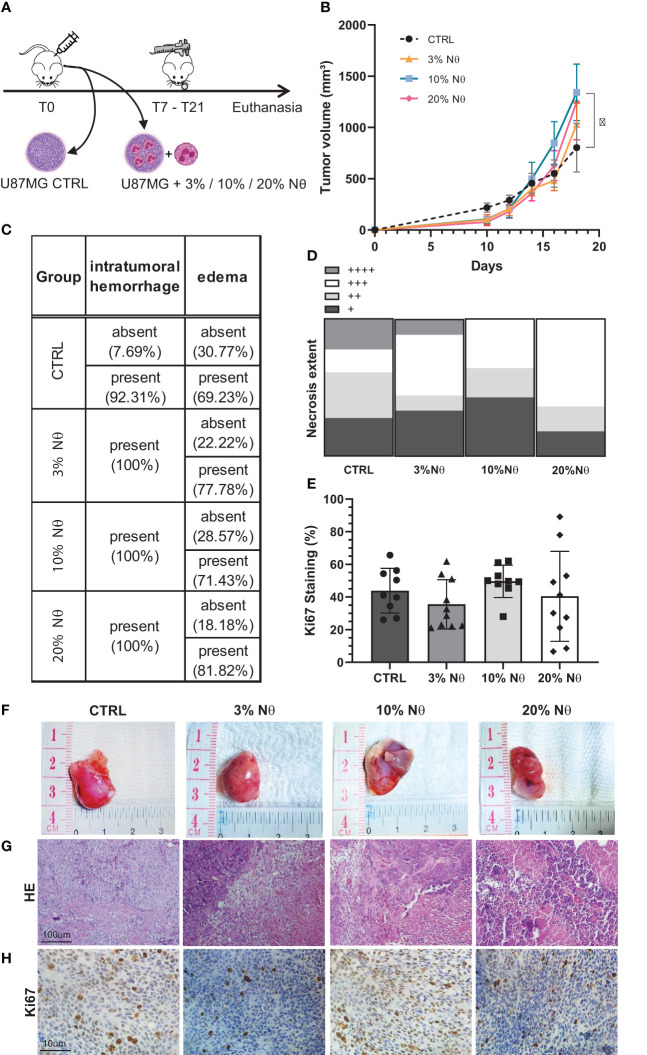
**(A)** *In vivo* glioblastoma protocol. **(B)** Tumor growth measured by day. **(C)** Histopathological quantitative analysis. **(D)** Necrosis extent quantification. **(E)** Ki67 staining percentage. **(F)** Representative images from tumor at euthanasia, **(G)** from HE staining, and **(H)** Ki-67. Two-way ANOVA, multiple comparisons. Tukey test, in which a = 0.05, *P < 0.05. Error bars are mean ± SD.

To confirm our results *in vivo*, we injected, subcutaneously, 10^6^ U87MG cells along with increased proportions of neutrophils (3%, 10%, and 20%) into the right flank of athymic BALB/c nude mice (**Figure 3A**). **Figure 3B** shows the tumor volume of each group. Notably, CTRL tumors (only U87MG cells) have a slightly bigger initial growth than other groups (with neutrophils), however, the tumorigenesis pattern shifts after day 18, in which 10% of the neutrophil groups emerged above control (*P* < 0.05). Despite the use of both male and female BALB/c nude mice, the histopathology showed no differences between sex regarding intratumor hemorrhage and edema (**Figure 3C**), necrosis extent (**Figure 3D**), and Ki67 staining (**Figure 3E**). In addition, no neutrophil infiltration was detected in all experimental groups analyzed following 20 days of tumor implant (**Supplementary Table 3**). Although tumor proliferation did not present statistically significant difference, the 10% neutrophil group seemed to be concentrated in around 50% of Ki67 staining while the other groups have a broader distribution. Representative images of tumor size (**Figure 3F**), HE staining (**Figure 3G**), and Ki67 staining (**Figure 3H**) are displayed according to each group. Overall, the given data suggest that tumor-associated neutrophils induce a significative, but modest, *in vivo* tumor progression using the preclinical model of glioblastoma in nude mice. It is probably that an integral immune system interaction is required to neutrophils drive effective *in vivo* tumor growth.

### Energy metabolism of glioma cells shifts due to glioma-neutrophil interactions

**Figure 4:**
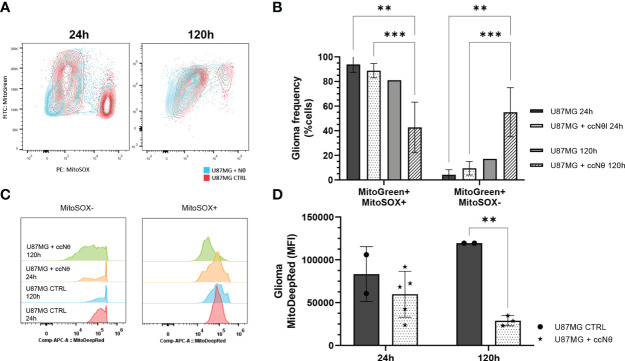
Evaluation of mitochondrial function by flow cytometry mitochondrial probes MitoTracker Green, MitoTracker Red and MitoSOX Red. **(A)** Gating strategy overlaps between groups. **(B)** Quantification of mtROS production from selected MitoTracker Green+ cells. **(C)** MitoTracker DeepRed MFI between groups. **(D)** Quantification of MFI in U87MG cell line with or without neutrophils for 24 h and 120 h. Two-way ANOVA, multiple comparisons. Tukey test, in which a = 0.05, **P < 0.005 and ***P < 0.001. Error bars are mean ± SEM.

Another set of experiment addressed the energy metabolism of glioma cells in the presence and absence of neutrophils and, thus, we evaluated tumor mitochondria functionality using mitochondrial probes. The results indicate that glioma cells sustain the number of mtROS (mitochondrial reactive oxygen species) production in the first 24 h, followed by a decrease after 120 h of co-culture with neutrophils (*P* < 0.005; **Figures 4A, B**). Meanwhile the opposite effect was observed for mtROS negative clustering as it increases after 120 h of co-culture with neutrophils (*P* < 0.005). MitoTracker DeepRed indicated a sharp disruption of the mitochondrial membrane potential after 120 h of co-culture with neutrophils (*P* < 0.005; **Figures 4C, D**). Altogether, this data implies glioma mitochondrial recovery after culture with neutrophils for 120 h, along with a reduction of membrane potential in the presence of neutrophils, indicating dysfunctional organelles.

### Glioma-neutrophil crosstalk results in the release of an immunosuppressive secretome

**Figure 5:**
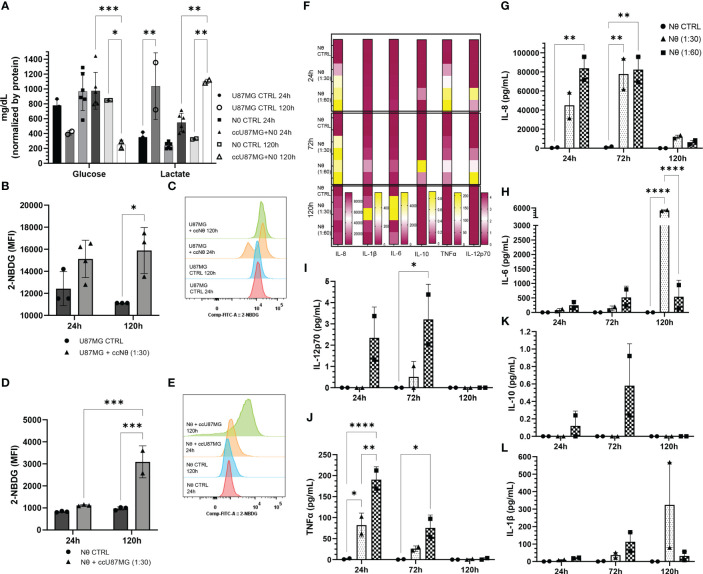
**(A)** Quantification of glucose consumption and lactate production from co-culture (cc) supernatant compared to control in 24 h and 120 h. Data normalized by protein. **(B)** 2-NBDG uptake assay: U87MG quantification **(B)** and MFI **(C)**; and neutrophil quantification **(D)** and MFI **(E)**. Cytokines pattern release in co-culture supernatant express in heat map **(F)** and quantification of IL-8 **(G)**, IL-6 **(H)**, IL-12p70 **(I)**, TNFα **(J)**, IL-10 **(K)**, and IL-1β **(L)**. Two-way ANOVA followed by Bonferroni & Šidák corrections for multiple comparisons, in which α = 0.05, *P < 0.05, **P < 0.005, ***P < 0.001, and ****P < 0.0001. Data shown as mean ± SEM.

To explore factors modulating the cell-to-cell communication in the glioma-neutrophil crosstalk soluble factors, we assessed the release by either the glioma cells or by the neutrophils. First, glucose and lactate production were evaluated in culture media after 24 h and 120 h of culture (**Figure 5A**). The bar graph shows great use of glucose and, consequently, higher production of lactate over time, especially regarding glioma-neutrophil culture. We then analyzed glucose-uptake, which revealed that glioma cells have a slight glucose uptake increase after 24 h of co-culture with neutrophils, followed by a complete cell recovery after 120 h (**Figures 5B, C**). Similarly, neutrophils appeared to increase their glucose-uptake after 120 h of co-culture with glioma cells as well (**Figures 5D, E**), indicating that glucose consumption by both cells contributes to lactate production. Additionally, a Human Inflammatory Cytometric Bead Array (CBA) was conducted to determine the *in vitro* cytokine production of glioma-neutrophil culture over time and with different proportions of neutrophils (1:30 or 1:60). *In vitro* cytokine production showed three different states that can be clustered by culture duration (**Figure 5F**). In the first 24 h, glioma-neutrophil cultures produced high amounts of IL-8 and TNFα, slightly increased amounts of IL-12p70, and low to zero levels of IL-1β, IL-6, and IL-10. After 72 h, we identified a proportional increase of tested cytokines, especially with regards to IL-8, IL-10, and IL-12p70, while the production of TNFα was reduced. However, after 120 h, this pattern shifted and there was a rise in IL-1β and IL-6 release, while IL-8 declined and there was almost no IL-10, TNFα, and IL-12p70 production. All control neutrophils, regardless of culture duration, showed very low or no cytokine production. A detailed quantification and analysis demonstrated a significant IL-8 production of glioma-neutrophil co-cultures after 24 and 72 h, with a sharp downturn after 120 h (**Figure 5G**). The opposite was observed for IL-6 in glioma-neutrophil (1:30) experimental group, in which IL-6 production was 10 times higher (**Figure 5H**). Despite only detecting low levels of IL-12p70, its expression pattern is comparable with IL-8, showing increased levels in the first hours, followed by basically absent production after 120 h (**Figure 5I**). Differently from IL-6, the antithesis of the production of TNFα had a high initial expression, proportionally decreasing over the hours (**Figure 5J**). Finally, there were no significant alterations in IL-10 and IL-1β production (**Figures 5K, L**). Overall, the data suggest that the neutrophil-glioblastoma crosstalk potentiates the differential release of immune cell recruiting and suppression molecules described as drivers of tumor progression.

### Glioma cells reprogram cellular functions of neutrophils

**Figure 6:**
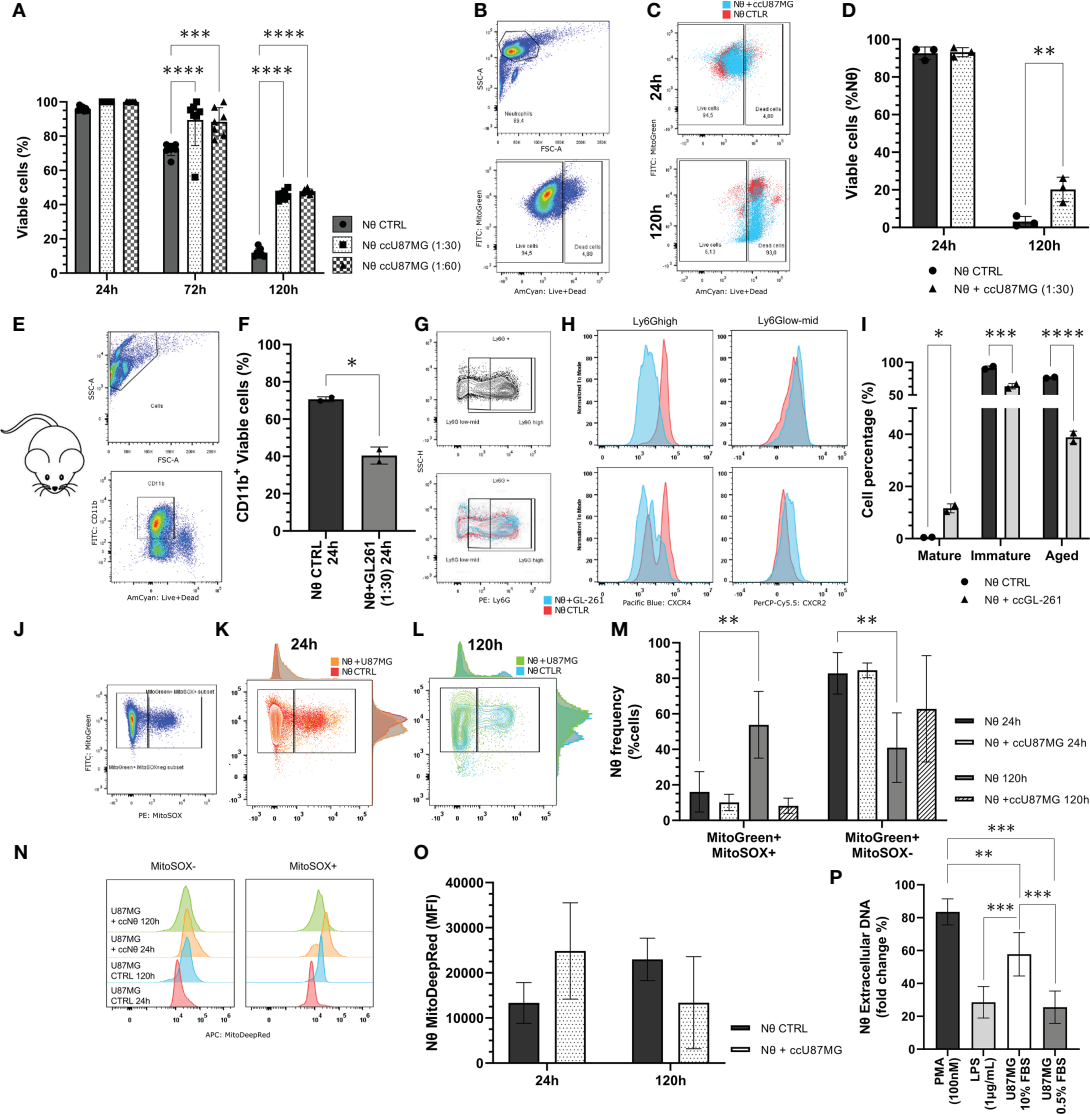
**(A)** Neutrophil viability tests performed using the trypan blue dye exclusion test throughout 24 h, 72 h and 120 h of co-culture along with U87MG (ccU87MG) in different proportions (1 U87MG: 30/60 Nq). **(B)** Gating strategy to analyze neutrophil viability. **(C)** Live/Dead overlap between experimental groups. **(D)** Percentage of live neutrophils. **(E)** Gate strategy to select CD11b+ cells **(F)** Cell viability quantification from neutrophil derived from mice C5/BL6 bone marrow isolation. **(G)** Gating strategy to select Ly6G populations. **(H)** MFI for CXCL4 and CXCL8. **(I)** Quantification of neutrophil maturation state according to mature: Ly6Ghigh, CXCR4-, CXCR2+; immature: Ly6Glow/mid, CXCR4+, CXCR2low; and aged: Ly6Ghigh, CXCR4+, CXCR2-. **(J)** Gating strategy to cluster MitoGreen/MitoSOX **(K)** in 24 h and **(L)** 120 h. Evaluation of neutrophil mitochondrial function by flow cytometry mitochondrial probes MitoTracker Green, MitoTracker Red, and MitoSOX Red. **(M)** Quantification of the percentage of mitochondrial ROS production in neutrophils with or without U87MG, for 24 h and 120 h. **(N)** MitoTracker DeepRed MFI distribution and **(O)** quantification. **(P)** NETs formation observed after 3h incubation with SytoxGreen reagent. Two-way ANOVA, multiple comparisons, Tukey test, in which a = 0.05, *P < 0.05, **P < 0.005, ***P < 0.001, and ****P < 0.0001.

In addition to the analysis of effects of the neutrophil-glioma crosstalk on the tumor cells, we also evaluated alterations in the neutrophil population. Staining with trypan-blue, along with an analysis by flow cytometry, revealed that the co-culture of neutrophils and U87MG increased the neutrophil life span when compared with neutrophils cultured in absence of tumor cells (**Figure 6A-C**). The amount of live neutrophils in the presence of glioma significantly increased, considering the period of 120 h (**Figure 6D**). In addition to experiments with human cells, we extracted neutrophils from murine bone marrow and cultured them with murine GL-261 cells for 24 h (**Figure 6E**). This co-culture resulted in a reduction of neutrophil (CD11b^+^) viability (*P* < 0.05; **Figure 6F**). However, different maturation states can be observed when murine neutrophils were challenged with GL-261. With this regard, we detected an increase of over 10% of mature neutrophils, after co-culture with GL-261 (*P* <.05), and significant decreases of the immature (*P* < 0.001) and of aged population (*P* < 0.0001; **Figures 6G–I**). Furthermore, we evaluated neutrophil mitochondria functionality with mitochondrial probes. Gating strategy for MitoTracker Green^high^ MitoSOX red ^-/+^ (**Figure 6J**) demonstrated different neutrophil populations after 24 h (**Figure 6K**) and 120 h (**Figure 6L**), which indicates a substantial amount of MitoSOX^-^ neutrophils when cultured with glioma cells (**Figure 6M**). Moreover, the data illustrates no significant alterations in mitochondrial membrane potential (**Figure 6N, O**). Concerning NETs release, neutrophils were treated with conditioned medium harvested from cultured U87MG cells and compared with commonly used chemicals (PMA 100 nM and LPS 1 μg/mL) for 3 h. Data revealed that glioma soluble factors were able to induce neutrophil DNA extravasation (*P* < 0.005; *P* < 0.001; **Figure 6P**). Altogether, the data have demonstrated the great ability of tumors to modulate the life span and response of neutrophils.

### Cytokines play an important role in recruiting neutrophils and sustaining anti-inflammatory state

**Figure 7:**
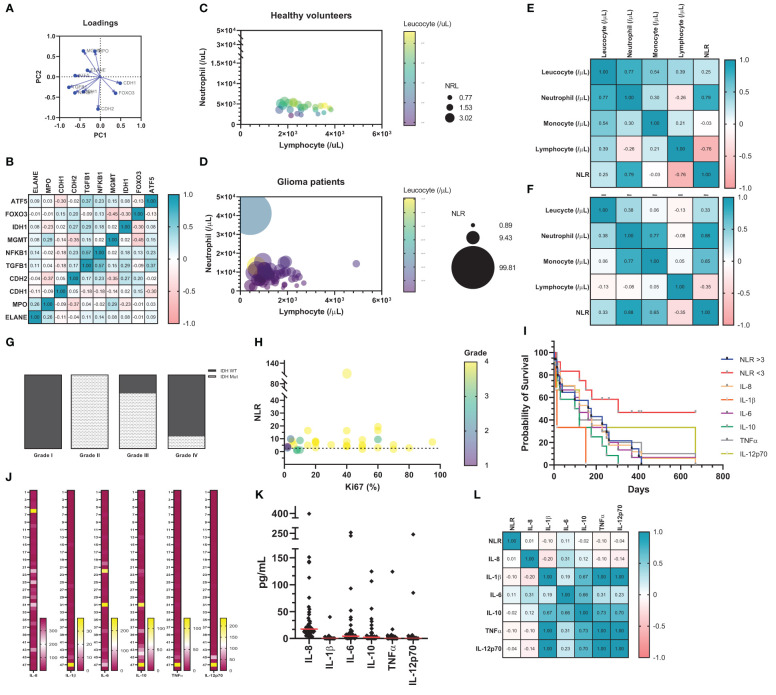
**(A)** Data download from TCGA GBM and analyzed by XENA browser (n = 172). The principal component analysis (Loadings plot) **(B)** Pearson’s correlation matrix test, in which the strength of association is indicated. Correlation analysis between Neutrophil, Lymphocyte, Leucocyte, and NLR from healthy volunteers **(C)** and glioma patients **(D)**. On this graph, about 20 healthy volunteers and 50 glioma patients are shown as individual circles. The X-coordinate of each circle represents circulating neutrophils/μL, whereas the Y-coordinate represents the circulating lymphocyte/μL. The size of each symbol is proportionate to the NLR. Finally, the color of each symbol represents the total circulating leucocyte/μL. Correlation matrix (Pearson’s coefficient) indicates strength of association from patients database (n = 20 healthy **(E)**; n = 50 GB patients **(F)**. **(G)** IDH status stratify according to grading system. **(H)** Correlation analysis between NLR, Ki67 staining and grade from glioma patients. On this graph, about 50 glioma patients are shown as individual circles. The X-coordinate of each circle represents circulating Ki67 staining in percentage (%), whereas the Y coordinate represents the NLR, in which the dashed line represents the limit between the ratio considered normal and high (NLR = 3.0). The size of each symbol is proportionate to tumor grade (I – IV). **(I)** Kaplan-Meier survival analysis. Cytokine quantification in heat map **(J)** and dot plot **(K)**. **(L)** Heat map with correlation analysis between NLR and cytokine from blood serum of glioma patients.

To validate a potential role of neutrophils in clinical samples, a principal component analysis was performed (Loadings plot, **Figure 7A**). The results showed that the variables CDH1 and FOXO3 are clustered closely together, indicating that these two variables are positively correlated, similar to MGMT and MPO and to IDH1, TGFβ1, and NFκB1. In comparison, the vectors for FOXO3 and MGMT form a nearly 180° angle, indicating that these variables are negatively correlated; the same was observed with CDH1 and ELANE. Finally, the vectors for variables FOXO3 and CDH1 form a nearly right angle with IDH1, TGFβ1, and NFκB1, suggesting that these variables are likely uncorrelated. Overall, the data suggests that neutrophils act in accordance with immunosuppressive signaling pathways, especially with MGMT (**Figure 7B**). In our patient cohort, we performed a correlation analysis between neutrophils, lymphocytes, leucocytes, and neutrophil-lymphocyte ratio (NLR) in healthy volunteers (**Figure 7C**) and glioma patients (**Figure 7D**). This analysis illustrated a considerable shift in blood count in glioma patients. The results imply an important neutrophil-monocyte correlation improvement in glioma patients (**Figures 7E, F**), which can explain the role of innate cells in sustaining immunosuppressive TME in glioma. To sum up, a descriptive analysis of glioma patient dataset shows a considerable amount of glioblastoma grade IV (n = 27), notably with IDH wild-type (n = 19) (**Supplementary Table 4**). Patients IDH status was stratified according to grading system to visualize the existence of any condition related to IDH (**Figure 7G**). Furthermore, the amount of circulating neutrophil significantly increased in glioblastoma patients, when compared with non-glioblastoma **(** **Supplementary Table 4** - P < 0.0001). The correlation analysis between NLR, Ki-67 staining, and grade from glioma patients was performed to test the connection between those parameters. This graph shows no correlation between NLR and Ki-67 staining, the dashed line represents the limit between the ratio considered normal (NLR = 3.0; **Figure 7H**). Afterwards, the Human Inflammatory CBA was conducted in blood serum from glioma patients and the Kaplan-Meyer survival graph was used to analyze the data (**Figure 7I**). The data indicates poor prognostic value of NLR >3, IL-1β, and IL-10, which validates our *in vitro* results described above. Cytokines from each patient was stratified to understand the big picture, the last one (n° 48) represents the healthy subject (**Figure 7J**). Quantified data shows high amount of serum IL-8, which is consistent with neutrophil mobilization, recruitment, and tumor infiltration. IL-6 and IL-10 also showed considerable amounts, which converses with the immunosuppressive state (**Figure 7K**). Pearson’s test demonstrated correlation between serum IL-1β, IL-10, TNFα, and IL-12p70; while IL-10 is also closely related to IL-8 (**Figure 7L**). Overall, the given data suggests a cytokine milieu in glioma patients that favors recruitment of neutrophils, sustaining an immunosuppressive profile, that which is associated with poor prognosis.

## Discussion

The intercellular interaction and communication within the tumor is crucial for understanding the TME (28, 29). The tumor niche includes, in addition to tumor and stromal cells, fibroblasts, immune, and endothelial cells (5, 30). The immune infiltrate, including neutrophils, is a feature of chronic inflammation, contributing to tissue damage and ultimately to tumor progression (13). In this context, we were able to demonstrate that neutrophils act as expected in the first 24h after being in contact with glioma cells: they attack tumor cells and compromise their viability. This action persists until the next hours, and after 72 h it is possible to observe a slight change in this behavior, suggesting that from that point on, tumor cells are successful in reprogramming the functions of neutrophils. The data suggest an important role of neutrophils in tumor progression, both *in vitro* and *in vivo*. The neutrophil plasticity happens according to the stimuli received, and time seems to be a major factor for their reprogramming. Other authors as well have presented evidence supporting this theory (13, 31–33). After 120 h, we observed that the tumor reacts to the presence of the neutrophils and its viability increases. This behavior shows that the “contact time” factor is a variable that must be considered when studying a neutrophil response in tumor progression. It also indicates that tumor-infiltrating neutrophils may have heterogenous functions depending on their actual time in the TME.

The results of Ki-67 labeling demonstrated that the neutrophil sphere groups proliferated almost exclusively in the periphery – consistent with the literature and with clinical findings in glioma patients (34) – since this region has greater access to nutrients and oxygen, while the central area shows necrosis. Additionally, while the control sphere, U87MG, revealed Ki-67 positivity of about 40%, the neutrophil groups, in both initial and advanced tumor 3D models, reached a plateau of about 80%, demonstrating that growth was significantly higher when there is crosstalk between these cells. Necrosis is a relevant finding in clinical tumor samples, being an indication of malignancy (35). Thus, since our experiments revealed increased levels of necrosis in the presence of neutrophils, it is suggested that neutrophils could help in inducing necrosis within the tumor niche, favoring the poor prognosis in glioma patients. Moreover, tumor infiltration of neutrophils has been described as an indicator of poor prognosis (36) and the location within the tumor mass has prognostic relevance, since they are usually found in the peritumoral area (23, 37, 38). Our *in vitro* data suggests that neutrophils can attach to glioma sphere at the periphery (data not shown), similar to studies which have described mostly peritumoral neutrophil infiltration in tumor patients (4, 39). These results suggest that the location of the immune infiltration in the tumor is of high prognostic importance and impacts on its malignancy, and that the presence of neutrophils in the intratumoral region is highly associated with cancer progression and growth.

Furthermore, glioma mitochondria metabolism indicates a possible mitochondria distress, which can result in mtDNA release. The tumor depends on mitochondrial mismatch in its tumor escape process (40, 41). According to cell energy necessity, the ATP production is increased in stress conditions, such as immune interaction, high oxidative phosphorylation (OXPHOS) activity, necrosis, and hypoxia (42). High ATP production need leads to mitochondria fusion (43), which can explain the low MFI in MitoTracker DeepRed after 120 h with neutrophils culture. However, in tumor cells, such as glioma, the ATP produced is mostly used as a signal, being extravasated *via* pannexin channels, activating purinergic receptors, such as P2Y_2_ in immune cells, and leading to the activation and chemotaxis of neutrophils (44). The Warburg effect can be observed by the high lactate production in the mixed culture. This effect describes the use of glucose preferentially in the glycolytic pathway, even in the presence of oxygen, which accelerates tumor metabolism and, consequently, lactate production (45, 46). Cell stress can initiate various signaling pathways (47). Our data implies the possibility of glioma mtDNA release into cytosol. Tumor cells can initiate NFκB signaling, increasing IL-8 and reducing IL-6 secretion, mtDNA can activate cyclic GMP-AMP synthase and inflammasome pathways. Mobilizing cGAS-STING1 and canonical NFκB signaling stimulates TNFα and IL-6 cytokine secretion, while inflammasome mechanism cleavage IL-1β and, together, activate immune responses (48–50). With this regard, our *in vitro* results suggest that in 24 h and 72 h there is an increase in IL-8 production in glioma cells challenged with neutrophils, which might be explained by the NFκB signaling. This converses with glioma viability, protein synthesis, and MTT absorbance. Neutrophils first interaction cause glioma distress, which has a turnover over the hours. The remaining living glioma cells might be able to activate cGAS-STING1 pathway and release TNFα. On the other hand, after 120 h, glioma mitochondria data indicates mitochondria recovery and fusion, which can be due to the increase in energy metabolism and ATP production necessity (43). Authors have evaluated ATP increase in distress glioma cells (46). Moreover, cell resurgence is in accordance with the increase in IL-6 and IL-1β *in vitro* production, after 120 h.

Neutrophils are formed from the common myeloid precursor in the bone marrow and circulate for a limited time, capable of being active for approximately 3 days after stimulation (13, 31, 51). The aging process of the cell is marked by the phenotypic activation triggered by some disturbance (52). Nonetheless, there is evidence that survival rate changes when there is cellular hyperresponsiveness; the mechanisms by which the life span is increased are still under investigation for different chronic inflammatory diseases (53–57). Notably, neutrophil modulation indicates onset by the third day, while the viability of U87MG in that same period suggests a break in the immune cell attack process against the tumor. In other words, a period of prolonged contact is necessary for neutrophils to effectively assist in tumor growth. Indeed, glioma mtDNA can activate neutrophil response *via* TLR9 (58, 59) leading to the increased release of inflammatory cytokines, including TNFα, IL-6 and IL-1β (58). Moreover, mtDNA can trigger NETs release (60).

Neutrophils are well-known for modulating their own metabolism under environmental circumstances (61). Neutrophils have few mitochondria; during maturation process, however, they have an increase in OXPHOS (62–64), describing a metabolic shift towards free fatty acids (FFAs)-dependent OXPHOS (65). Our data suggests that neutrophil mtROS production is decreased and probably not the major ROS source production in these cells. Oxidative neutrophils have an essential role in inhibiting T cell activation, especially in environments with limited glucose (61, 63, 66). Similar observations were made in our study. Both, tumor cells and neutrophils, increased glucose-uptake, with subsequent increase of lactate production. Moreover, high lactate production has been described to contribute to immunosuppression in the TME (67, 68).

Neutrophil azurophilic granules are rich in myeloperoxidase (MPO) and in proteolytic and bactericidal proteins, being directly involved with phagocytosis (69, 70). During neutrophil oxidative burst, MPO granules inside the cell generate HOCl. Those oxidants are essential to NETosis (71). NETs are a nuclear network composed of granular proteins such as elastase (NE) and MPO (72). This process is considered a mechanism of cell death different from apoptosis and necrosis since it consists of disintegrating the nuclear envelope, exposing the content in the cytosol. The detriment of the intercellular membrane leads to the loss of organelles, and, consequently, the integrity of the plasma membrane is impaired (73). DNA-binding proteins, such as High Mobility Group Box 1 (HMGB1), are increased in NETs-mediated activation of TLR4 and TLR9 (74). One study revealed that HMGB1 interaction with RAGE/NFκB receptors from glioma cells induces IL-8 expression in tumor cells (75). The data suggest a higher NETs release in the presence of glioma soluble factors. Under tumor conditions, NETs have been linked to the formation of metastases, because, theoretically, these bonds capture the circulating tumor cells and facilitate the disposition in other tissues (76, 77). Moreover, NETosis pathway requires ROS generation and increase in intracellular Ca^2+^ (78). This is mediated by mitochondria ATP production which sustain a positive feedback loop specially by activating purinergic receptors, such as P2Y_2_ (44). Although the NETs release is a cell death mechanism, studies have demonstrated that some neutrophils remain alive after NETosis due to the chromatin source, which is mitochondrial (79). Therefore, it is suggested that GB-neutrophil crosstalk have a prolonged tumor activation that could involve NETs formation and the release of HMGB1, MPO and NE proteins by neutrophils, which activates glioma cells by NFκB signaling, inducing oxidative mutations, impairing mitochondrial function and secreting immunosuppressive cytokines.

One study observed in a zebrafish glioblastoma model that neutrophils are recruited very early during oncogenesis and their presence increases tumor cell proliferation. The study suggests that this is due to the release of ROS, capable of causing DNA damage and inducing oncogenesis in cells around the tumor (80). We observed in our *in vivo* experiments that neutrophils can shift tumor growth pattern and increase its size along time. However, the animal model revealed that neutrophil alone have low effect in the overall outcome, BALB/c nude mice have no adaptive immune system, and the implantation of human neutrophils might not have the strength to recruit mice innate cells, such as monocytes. A recent study demonstrated the ability of neutrophils to recruit macrophages and regulatory T cells in hepatocellular carcinoma *via* cytokine release (81). Moreover, our clinical dataset demonstrated a strong positive correlation between circulating neutrophils and monocytes in GB patients. Our patients dataset revealed that high-grade glioblastoma had Ki-67 positivity increased by almost two times when compared with non-glioblastoma, in accordance with higher NLR (>3). Studies have demonstrated that higher percentage of Ki-67-positive cells in gliomas is related to the higher concentration of neutrophils in relation to lymphocytes in peripheral blood. Furthermore, this proportion is considerably higher in grade IV tumors (82). Overall, the data suggest a relevant crosstalk between inflammation and Ki-67 in glioma, corroborating our findings in the U87MG spheres, in *in vivo* BALB-c nude mice and clinical samples, in which there is a propensity between cell proliferation in control group and in groups with neutrophils.

It is not unusual to find pro-inflammatory cytokines in the TME, acting as stimulators of tumor progress and metastasis (83). Cytokines such as TNFα and IL-1β – known for their generally pro-inflammatory functions – and IL-10, a cytokine with immunosuppressive capacity are often found in the TME (84). Within the TME, some of the main cytokine-producing cells are B and T lymphocytes, dendritic cells, tumor-associated macrophages, TANs, MDSCs, and NK cells (85–87). Furthermore, IL-6, IL-8, and IL-10 production by cells from the TME mediate pro-tumor functions of immune cells, such as neutrophils and macrophages (88, 89). Our data demonstrates that glioma patients present an important cytokine-related worse outcome, especially regarding IL-1β and IL-10. Notably, the information provided by the release of cytokines in patients with glioma corroborates those found *in vitro*. In general, there is a high amount of circulating IL-8, in the same way that there is an increase in gliomas challenged with neutrophils. We found this pattern of late release of IL-1β and IL-6, which shows to be related to a worse prognosis in our patients. Moreover, the importance of IL-10, being preferentially released by immune cells, in this case, neutrophils, as indicated in the literature (84, 90–92). These data together with the NLR indicate great relevance of the role of neutrophils in both local and systemic immunosuppression.

**Figure 8:**
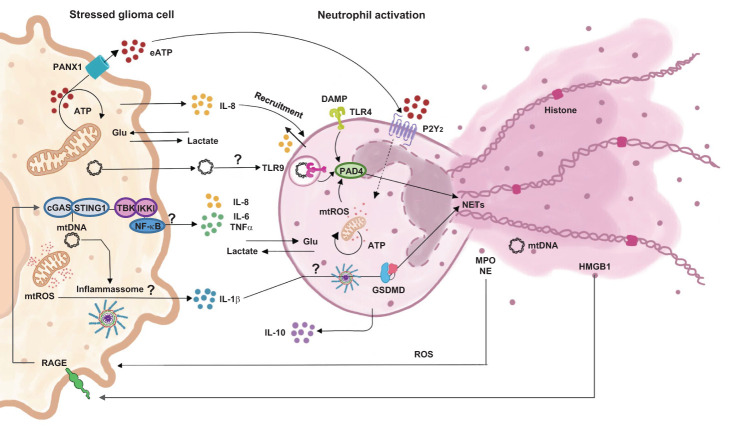
Proposition of cell signaling pathways involved in dual communication between glioblastoma and neutrophils.

Overall, our study answers an important question about the ability of dual communication followed by cell modulation between glioma and neutrophils (**Figure 8**). Bringing out the important role of neutrophil immunosuppression activation. Briefly, we have shown that, when cells are in contact, there is a constant and time dependent modulation. With tumor activation by mitochondrial disruption and mtROS release. The set of cytokines released is consistent with the maintenance of an immunosuppressed environment, which would explain the important role of this communication for the perpetuation of a TME favorable to the tumor. Due to cellular activation, both cells consume glucose, which leads to an increase in lactate in the environment and potentiates the effects of immune inhibition. On the other hand, pathways yet to be discussed activate neutrophils, promoting the release of NETs and ROS, which in turn contribute to the maintenance of the chronic inflammatory state found in the TME. It is important to emphasize that the activation pathways that lead to these signals need to be better elucidated. New experiments should be carried out to try to enlighten the open questions raised in our study about the signaling pathways between glioma-neutrophil crosstalk.

## Concluding remarks

Thus, the data demonstrates a bidirectional crosstalk between glioma cells and neutrophils in modulating not only each other but also the local microenvironment. This study allowed insights into the complex interaction between tumor cells and the microenvironment that surrounds them. Briefly, we observed that the presence of neutrophils closely located to the tumor is capable of inducing tumor-promoting effects. We have verified that there is a glioma-neutrophil crosstalk, resulting in further increase of neutrophil recruitment as well as elongation of neutrophil lifespan. These results are relevant to understand how tumor progression occurs and how immune cells contribute to that process. However, further studies are needed to confirm the magnitude of this interaction.

## Data availability statement

The original contributions presented in the study are included in the article/**Supplementary Material**. Further inquiries can be directed to the corresponding author.

## Ethics statement

The studies involving human participants were reviewed and approved by Irmandade Santa Casa de Misericórdia de Porto Alegre. The patients/participants provided their written informed consent to participate in this study. The animal studies were reviewed and approved by Hospital de Clınicas de Porto Alegre, UFCSPA, and USP.

## Author contributions

DR and EB contributed to the study conception and design. DR, NO, PS, MA, PB, LS, LD, NG, AR, GL, AMA, GK, AP, FV, ML, MW, PW, ABA, JS, NC, NL performed material preparation, data collection and/or analysis. DR wrote the first draft of the manuscript. EB supervised the study and granted financial support. All authors commented on previous versions of the manuscript. All authors have read and approved the final manuscript.

## Supplementary material

Supplementary Figure 1 Analysis of neutrophil infiltration in U87MG spheroids by CD45 immunohistochemistry. Representative images from **(A)** U87MG sphere; **(B)** U87MG + N0 sphere; **(C)** U87MG + pool N0 sphere for 72h; **(D)** U87MG + pool N0 sphere for 120 h (magnification 40x).

Supplementary Table 1 List of materials.

Supplementary Table 2 3D histopathological analysis.

Supplementary Table 3 Histopathological analysis of HE stained slices of glioblastomas implanted in Balb/c male and female nude mice.

Supplementary Table 4 Glioma patients group description. *anaplastic astrocytoma, metastatic adenocarcinoma, transitional meningioma, “no evidence of neoplasia in histological findings,” pilocytic astrocytoma, oligodendroglioma, diffuse Large B Cell Lymphoma, and “astrocytoma” – all described as per medical report.
