# Supplementary figures and images for: Tumor-neutrophil crosstalk promotes in vitro and in vivo glioblastoma progression
Source: Front Immunol. 2023 May 24;14:1183465. doi: 10.3389/fimmu.2023.1183465 (PMC10244780; doi:10.3389/fimmu.2023.1183465)

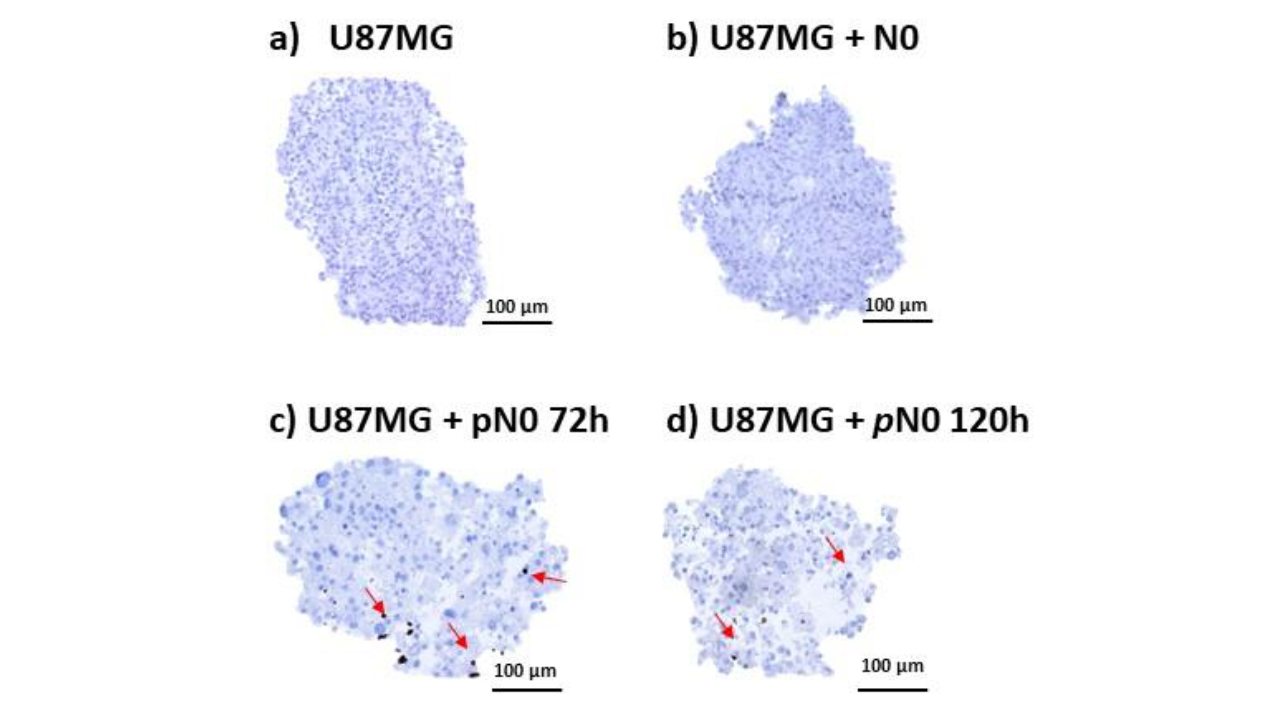

Supplement: Supplementary file 1 [file Image_2.tif]

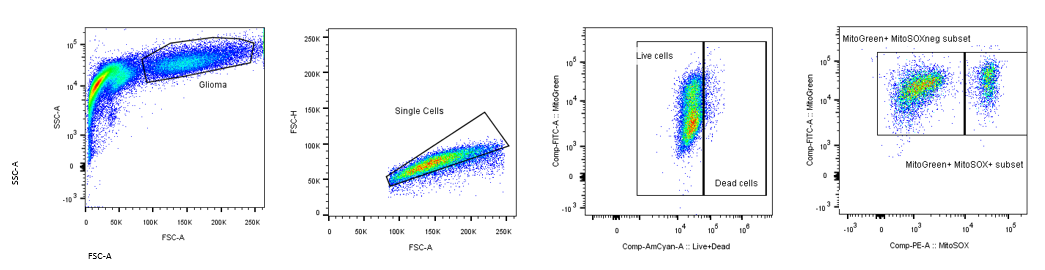

Supplement: Supplementary Figure 1 — Analysis of neutrophil infiltration in U87MG spheroids by CD45 immunohistochemistry. Representative images from (A) U87MG sphere; (B) U87MG + N0 sphere; (C) U87MG + pool N0 sphere for 72h; (D) U87MG + pool N0 sphere for 120 h (magnification 40x). [file Image_1.tif]
