# Supplementary material for: Tumor-neutrophil crosstalk promotes in vitro and in vivo glioblastoma progression
Source: Front Immunol. 2023 May 24;14:1183465. doi: 10.3389/fimmu.2023.1183465 (PMC10244780; doi:10.3389/fimmu.2023.1183465)
Supplement: Supplementary Table 1 — List of materials. [file Table_1.docx]

**Supplementary tables**:

Table S1: List of materials

| **Reagent or Resource** | **Source** | **Identifier** |
| --- | --- | --- |
| **Chemicals** | | |
| DMEM-Dulbecco's modified Eagle's medium | Gibco™ (Gibco BRL, Gaithersburg, USA) | 31600-34 |
| HEPES - 4-(2-Hydroxyethyl)piperazine-1-ethanesulfonic acid, N-(2-Hydroxyethyl)piperazine-N′-(2-ethanesulfonic acid) | Sigma-Aldrich (Sigma Chemical C0., ST. Louis, USA) | 7365-45-9 |
| Sodium bicarbonate (NaHCO3) | Neon Comercial Ltda, BRL | 1715 |
| Fetal bovine serum (FBS) | Gibco™ (Gibco BRL, Gaithersburg, USA) | 12657-029 |
| Fungizone | Gibco™ (Gibco BRL, Gaithersburg, USA) | 15290-018 |
| Penicillin/streptomycin | Gibco™ (Gibco BRL, Gaithersburg, USA) | 15140-122 |
| Dextran from Leuconostoc spp. | Sigma-Aldrich (Sigma Chemical C0., ST. Louis, USA) | 31389 |
| Histopaque®-1077 | Sigma-Aldrich (Sigma Chemical C0., ST. Louis, USA) | 10771 |
| MTT - (3-(4,5-Dimethylthiazol-2-yl)-2,5-Diphenyltetrazolium Bromide) | Sigma-Aldrich (Sigma Chemical C0., ST. Louis, USA) | M2128 |
| SRB \| Sulforhodamine B | Merck KGaA, Darmstadt, Germany | S1402 |
| Acetic Acid, Glacial | Merck KGaA, Darmstadt, Germany | A6283 |
| Tris base \| Tris(hydroxymethyl)aminomethane (10mmol/L pH 10,5) | Merck KGaA, Darmstadt, Germany | 252859 |
| Trypan Blue Solution, 0.4% | Gibco™ (Gibco BRL, Gaithersburg, USA) | 15250061 |
| Agarose | Merck KGaA, Darmstadt, Germany | A9539 |
| Paraformaldehyde | Merck KGaA, Darmstadt, Germany | P6148 |
| Hematoxylin monohydrate | Merck KGaA, Darmstadt, Germany | 3971 |
| Yellowish eosin | Synth, Diadema, Brazil | 45380 |
| Hydrogen peroxide solution | Merck KGaA, Darmstadt, Germany | 107298 |
| BSA \| Albumin bovine serum | Sigma-Aldrich (Sigma Chemical C0., ST. Louis, USA) | A7906 |
| Matrigel | Sigma-Aldrich (Sigma Chemical C0., ST. Louis, USA) | E1270 |
| SYTOX™ Green Ready Flow™ | Invitrogen \| Thermo Fisher Scientific Inc. (Waltham, USA) | R37168 |
| PMA \| Phorbol 12-myristate 13-acetate | Merck KGaA, Darmstadt, Germany | P1585 |
| LPS \| Lipopolysaccharides | Sigma-Aldrich (Sigma Chemical C0., ST. Louis, USA) | L3024 |
| **Antibodies** | | |
| LIVE/DEAD™ Fixable Dead Cell Stain Sampler Kit (1:400) | Thermo Fisher Scientific Inc. (Waltham, USA) | L34960 |
| Anti-Ki67 (1:100) | Dako \| Agilent (Santa Clara, USA) | M7240 |
| FITC Annexin V-PE Propidium iodide Apoptosis Detection Kit | BD Biosciences Pharmingen (San Diego, USA) | 555670 |
| Anti-CD11b \|M1/70 (1:400) | BioLegend (San Diego, USA) | 101206 |
| Anti-Ly6G \| 1A8 (1:400) | BioLegend (San Diego, USA) | 127608 |
| Anti-CD184 (CXCR4) \| L276F12 (1:100) | BioLegend (San Diego, USA) | 146511 |
| Anti-CD182 (CXCR2) \| TG11/CXCR2 (1:100) | BioLegend (San Diego, USA) | 129103 |
| **Probes** | | |
| MitoTracker Green | Invitrogen \| Thermo Fisher Scientific Inc. (Waltham, USA) | M7514 |
| MitoTracker Deep red | Invitrogen \| Thermo Fisher Scientific Inc. (Waltham, USA) | M46753 |
| MitoSOX | Invitrogen \| Thermo Fisher Scientific Inc. (Waltham, USA) | M36008 |
| 2-NBDG (2-(N-(7-Nitrobenz-2-oxa-1,3-diazol-4- yl)amino)-2-Deoxyglucose) | Invitrogen \| Thermo Fisher Scientific Inc. (Waltham, USA) | N13195 |
| **Critical Commercial Assays** | | |
| GLICOSE Liquiform | Labtest Diagnóstica S/A (BRL) | 133 |
| Enzymatic system for lactate | Labtest Diagnóstica S/A (BRL) | 138 |
| CBA - Cytokine cytometric bead array | BD Biosciences (San Diego, USA) | 552364 |
| EnVision™+ Dual Link System-HRP | Dako \| Agilent (Santa Clara, USA) | K4061 |
| Liquid DAB+ Substrate Chromogen System | Dako \| Agilent (Santa Clara, USA) | K3468 |
| **Solutions** | | |
| PBS 1x | 100mL \| Milli-Q water / 0,8g NaCl, 0,02g KCl, 0.115g Na_2_HPO_4_ e 0.02g KH_2_PO_4_ pH 7.4 | x |
| HBSS 10x | 1000mL \| Milli-Q water / 80g NaCl, 4g KCl, 0.6g Na_2_HPO_4_, 0.6g KH_2_PO_4_, 10g glucose | x |

Table S2: 3D histopathological analysis

| **Group** | **Mitosis (n°)** | **Necrosis** | **Descriptive analysis** |
| --- | --- | --- | --- |
| U87MG CTRL | 0 | - | fusiform cell shape |
|  | 4 | - |  |
|  | 0 | ++ |  |
|  | 4 | - |  |
| U87MG+Nθ | 1 | ++ | cavitaded |
|  | 1 | + |  |
|  | 0 | ++ |  |
|  | 0 | ++ |  |
| U87MG + 72h Nθ pool | 1 | + | small and homogeneous tumor cells / cavitaded |
|  | 0 | ++ |  |
|  | 1 | ++ |  |
|  | 1 | +++ |  |
| U87MG + 120h Nθpool | 0 | ++ | small and homogeneous tumor cells / cavitaded |
|  | 0 | ++ |  |
|  | 8 | - |  |
|  | 0 | ++ |  |
|  | 1 | ++ |  |

**Table S3**: Histopathological analysis of HE stained slices of glioblastomas implanted in Balb/c male and female nude mice.

| **Group** | **intratumoral hemorrhage** | **coagulative necrosis** | **necrosis extent** | **immune cell infiltration** | | **edema** | | **vascular proliferation** |  |
| --- | --- | --- | --- | --- | --- | --- | --- | --- | --- |
| Male CTRL | no | present | ++ | no | | no | | no |  |
|  | present | present | + | no | | no | | no |  |
|  | present | present | + | no | | present | | no |  |
|  | present | present | +++ | no | | present | | no |  |
|  | present | present | ++ | no | | present | | no |  |
|  | present | present | ++ | no | | present | | no |  |
|  | present | present | ++++ | no | | present | | No |  |
|  | present | present | + | no | | no | | No |  |
| Male 3% Nθ | present | present | +++ | no | | no | | No |  |
|  | present | present | +++ | no | | present | | No |  |
|  | present | present | + | no | | no | | No |  |
|  | present | present | ++++ | no | | present | | No |  |
|  | present | present | +++ | no | | present | | No |  |
| Male 10% Nθ | present (+++) | present | ++ | no | | present | | No |  |
|  | present | present | +++ | no | | present | | No |  |
|  | present | present | + | no | | no | | No |  |
|  | present | present | +++ | no | | present | | No |  |
|  | present | present | + | no | | no | | No |  |
|  | present | present | + | no | | present | | No |  |
|  |  |  |  |  | |  | |  |  |
|  |  |  |  |  | |  | |  |  |
| Male 20% Nθ | present | present | +++ | no | | no | | No |  |
|  | present | present | +++ | no | | present | | No |  |
|  | present | present | +++ | no | | present | | No |  |
|  | present | present | ++ | no | | present | | No |  |
|  | present | present | ++ | no | | present | | No |  |
|  |  |  |  |  | |  | |  |  |
|  |  |  |  |  | |  | |  |  |
| **Group** | **intratumoral hemorrhage** | **coagulative necrosis** | **necrosis extent** | **immune cell infiltration** | **edema** | | **vascular proliferation** | | |
| Female CTRL | present (+++) | present | ++ | no | present | | no | | |
|  | present | present | +++ | no | present | | no | | |
|  | present | present | +++ | no | present | | no | | |
|  | present | present | + | no | no | | no | | |
|  | present | present | ++ | no | present | | no | | |
|  | present | present | ++ | no | present | | no | | |
|  | present | present | + | no | present | | no | | |
|  |  |  |  |  |  | |  | | |
| Female 3% Nθ | present | present | + | no | present | |  | | |
|  | present | present | +++ | no | present | | no | | |
|  | present | present | + | no | present | | no | | |
|  | present | present | ++ | no | present | | no | | |
|  |  |  |  |  |  | |  | | |
| Female 10% Nθ | present | present | ++ | no | present | | no | | |
|  | present | present | + | no | no | | no | | |
|  | present | present | + | no | no | | no | | |
|  | present | present | ++ | no | present | | no | | |
|  | present | present | + | no | present | | no | | |
|  | present | present | +++ | no | present | | no | | |
|  | present | present | +++ | no | present | | no | | |
|  | present | present | +++ | no | present | | no | | |
| Female 20% Nθ | present | present | +++ | no | present | | no | | |
|  | no | present | + | no | no | | no | | |
|  | present | present | +++ | no | present | | no | | |
|  | present | present | + | no | present | | no | | |
|  | present | present | +++ | no | present | | no | | |
|  | present | present | +++ | no | present | | no | | |

Table S4: Glioma patients group description

| **Histopathological classification** | **Total number (n)** | **Sex**  **(mean age ±SD)** | **IDH (n)**** | | | **Ki-67 (%)** | **Neutrophil (/µL)** | **Lymphocyte (/µL)** | **NLR** |
| --- | --- | --- | --- | --- | --- | --- | --- | --- | --- |
|  |  |  | WT | Mut | Inconclusive |  |  |  |  |
| **Glioblastoma** | 27 | Women (57.9**±**21.1**)** | 19 | 3 | 5 | 45.4**±**24.2 | 9048.0**±**4428.3 | 1550.1**±**777.3 | 8.4**±**7.1 |
|  |  | Men (57.6**±** 17.3**)** |  |  |  |  |  |  |  |
| **Non-Glioblastoma*** | 16 | Women (60.3**±**14.2**)** | 6 | 3 | 1 | 25.6**±**28.8 | 5090.3**±**2707.0 | 1554.9**±**911.8 | 4.7**±**3.8 |
|  |  | Men (60.2**±** 11.5**)** |  |  |  |  |  |  |  |

*anaplastic astrocytoma, metastatic adenocarcinoma, transitional meningioma, “no evidence of neoplasia in histological findings,” pilocytic astrocytoma, oligodendroglioma, diffuse Large B Cell Lymphoma, and “astrocytoma” – all described as per medical report.
